# Integration Path Analysis of Traditional Media and New Media Based on Internet of Things Data Mining

**DOI:** 10.1155/2022/8193800

**Published:** 2022-05-06

**Authors:** Yinuo Liu

**Affiliations:** Liaocheng University, Liaocheng, Shandong 710016, China

## Abstract

With the development of information technology, the influence of traditional media is weakening day by day. In view of this, based on the Internet of things data mining technology, this study improves the k-means algorithm, and designs a new media precision marketing system, which combines new media with traditional media and provides a new marketing model for traditional media. The results show that the accuracy of the improved k-means algorithm finally reaches about 93%, which is much higher than that of similar algorithms. It can be seen that the improved k-means algorithm has better performance. In the application experiment, this study can effectively find the new media activities with the highest user preference, and the impact of the two websites' application of precision marketing system on users has also increased. It can be seen that the precision marketing system designed this time is more effective.

## 1. Introduction

The integration of new media and traditional media is an inevitable trend. On the one hand, it is driven by policies. As we all know, in recent years, traditional media have been greatly impacted by new media, resulting in the bankruptcy of paper media and brain drain. Therefore, the state strategically hopes and requires the traditional media to change in time and continue to dominate public opinion. On the other hand, facing the changes of media environment and users' reading and viewing habits, traditional media must also carry out self-revolution and media integration. In the era of mobile Internet, not only new channels, platforms, and products have been added, but people have been changed, demand, and consumption behavior have changed significantly, and the competitive logic of media has evolved.

With the development of modern information technology, the impact of traditional media industry on users is constantly challenged by new media technology. Users prefer fast and online media experience. In the past, the audience and market of traditional media were gradually severely squeezed (peiulis y.2021) [1]. This change in the media ecological environment has greatly weakened the advantages of traditional media such as newspapers, magazines, and television in channels and production resources, making it difficult for them to obtain the first opportunity in terms of real-time and entertainment in the process of new media competition. At the same time, compared with new media, the more institutionalized operation mode of traditional media industry is also lack of flexibility (Topbas b.2021) [2]. Using Internet of things, data mining technology and new media technology can help traditional media make up for their shortcomings and find a more feasible development model in the new media environment.

In order to make up for the shortcomings of traditional media in marketing mode, this study uses Internet of things data mining technology and new media technology to establish a precision marketing system for traditional media. The system is divided into three parts: data processing, business processing, and display system. Internet of things data mining and personalized recommendation are the main processing modules. The design of personalized recommendation module combines collaborative filtering algorithm, while the design of data mining module is based on the improvement of K-means algorithm. Particle swarm optimization algorithm is used to improve the selection effect of its initial clustering center, so as to achieve better data mining effect.

This study has two main innovations. The first innovation is to use particle swarm optimization algorithm to improve the k-means algorithm, improve its initial clustering center selection ability, and then make the improved k-means algorithm achieve better clustering effect. The second innovation is to use collaborative filtering algorithm and improved k-means algorithm to establish a new media precision marketing system, so that the model has the functions of data mining and personalized recommendation and overcomes the disadvantages of traditional media in marketing mode.

This study is mainly divided into four parts. The first part introduces the research background and existing research results in related fields, and leads to the research content of this study; the second part is the elaboration of the precision marketing model of new media; the third part is the demonstration and analysis of the experimental results of this study; and the fourth part is the conclusion of this study and the prospect of the future.

## 2. Related Works

Compared with traditional media, new media has higher flexibility and timeliness, richer channels, and stronger market advantages. Lu Jie and others investigated new media from three aspects: art and touch, art and tactile symbols, and tactile symbols in new media art. Finally, they proposed a new media research method with touch symbols as the core and a method of using art to benefit society (Lu *x* and others., 2019) [3]. The nazmine team collected data from 500 students through a questionnaire survey to understand how new media technology affects the interaction mode of Multan youth. Young people prefer to communicate through digital media technology, which increases their sense of isolation (nazmine and others. 2021) [4]. It combines advanced marketing concepts such as relationship marketing, viral marketing and event marketing, and puts forward a set of effective new media marketing strategies. The results show that new media marketing strategy can increase information coverage and interactivity, achieve more convenient communication, and improve marketing efficiency (MA t.2019) [5]. Wang *R* team conducted computer-aided interactive research on visual communication technology in new media and proposed an intelligent visual art creation form suitable for the development of the intelligent era. The results show that digital technology can improve the practical effect of digital media art from three aspects: visual expression, auditory expression, and audio-visual integration (Wang r.2021) [6].

As a mature clustering algorithm in the field of Internet of things data mining, K-means algorithm has been widely and deeply applied in all walks of life. Morais fo and others combined density functional theory calculation with K-means clustering algorithm to study the stability mechanism of eight atom binary metal clusters. The results show that the energy stability of binary clusters increases with the increase of the energy interval between the highest occupied molecular orbital and the lowest unoccupied molecular orbital (Morais fo and others. 2021) [7]. Sudarsono BG team determines the number and scale of scholarships through K-means clustering algorithm. The results show that this method more effectively divides students into four scholarship categories (Sudarsono BG and others. 2021) [8]. The method of mining voters by age (faminiag and others. 2029) and other types of social data was successfully applied by faminiah and others. Muhajir *m* and others used K-means clustering algorithm based on mixed data analysis between data observation and satellite imaging to generate more specific and up-to-date climate regions. Updated seasonal regions can provide more comprehensive data support for future government planning (muhajir *m* and others. 2021) [9]. Sintiya's team designed a hierarchical k-means algorithm by combining k-means algorithm with hierarchical method. The results show that this improvement can improve the selection ability of initial clustering centers of K-means algorithm and provide data support for policy zoning in poor areas (sintiya *s* and others. 2021) [10].

It can be seen that although the Internet of things data mining algorithm and new media technology have made great progress in recent years, there are few studies on the application of data mining algorithm to the integration of new media and traditional media, but traditional media is also an indispensable part of the current media ecological environment. Therefore, this study is based on the Internet of things data mining technology, and combines new media with traditional media to lay a foundation for the integrated development and new development of all kinds of media.

## 3. Establishment of New Media Precision Marketing Model for Traditional Media

### 3.1. Establishment of New Media Precision Marketing System Architecture for Traditional Media

**Figure 1:**
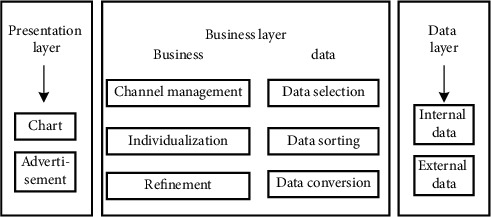
New media precision marketing system architecture.

**Figure 2:**
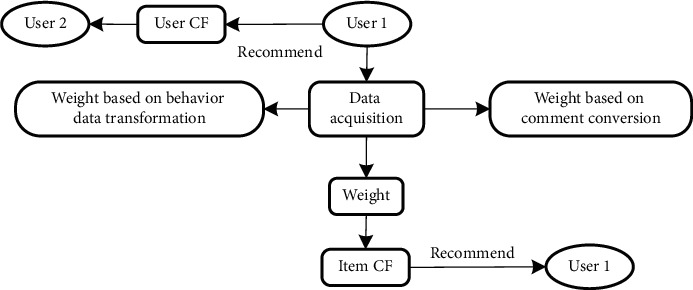
Processing flow of personalized recommendation module.

Great changes in the media industry have weakened the influence of traditional media, but the timeliness and accuracy of new media on the Internet of things can provide customers with personalized positioning and services, so as to help the traditional media industry segment and re-expand the consumer market (Qureshi IH and others. 2018) [11]. The establishment of traditional media precision marketing system is based on the marketing needs of traditional media, which can be divided into three categories: data acquisition and management needs, marketing business needs, and non-functional needs. The data collection and management requirements of the Internet of things are mainly user data and external data. User data is the basic attribute and behavior of users. In the architecture of most Internet companies, acquiring users is the work of the promotion and channel departments, and the next action is the user behavior to be performed by our operation. However, user conversion payment and user autobiography are the later behavior. Therefore, it is considered as the basic skill of user operation to make users active and retain users. Attributes such as geographical environment and time environment can represent user groups, while user characteristics such as attempt behavior and consumption preference can accurately locate user preferences. The combination of the two can extract the market outline of consumers; marketing business needs mainly include the distribution needs of market channels, user personalized recommendation needs, and implementation strategy needs. Non-functional requirements are mainly the security and performance requirements of the system. According to the above requirements, the framework of traditional media and new media precision marketing system established in this study is shown in Figure 1:

The system in Figure 1 is mainly composed of three parts: presentation layer, business layer, and data layer. The data layer is the most basic underlying system, that is, the data acquisition part, which is mainly divided into internal data acquisition and external data acquisition. The internal data is such as website data and software data, and the external data is mainly industry data and competitive data. By collecting and transforming internal and external data, you can build user's result labels. Prepare for the next personalized release, and adjust the external environment according to the needs of users. The business layer is mainly divided into business and data processing. Data processing uses data mining algorithm to process the data collected by the data layer. The business part realizes the specific business according to the processed data, that is, the segmentation of marketing channels, the formation of user personalized recommendations, and the fine management of core business. The main function of the presentation layer is to show the data collected and analyzed by the data layer and the business layer in the form of charts or personalized advertisements. It is the embodiment layer of the system. The order of the whole system from left to right is from the presentation link to the basic link. In the new media precision marketing system, the most important modules are two parts, one is the data mining module, and the other is the personalized recommendation module. In this study, in the personalized recommendation module, collaborative filtering algorithm is used to weight the processed user data, so as to judge the user's preference for goods, as shown in Figure 2:

Collaborative filtering algorithm is one of the most effective recommendation algorithms so far. It can be divided into heuristic and model-based algorithms according to different modes. This study adopts heuristic collaborative filtering algorithm (Han *x* and others. 2021) [12]. As shown in Figure 2, the heuristic collaborative filtering algorithm is divided into user-based cf (user CF) method and item-based cf (item CF) method. The system designed this time adopts the user CF method for personalized recommendation among the direct recommendations between users, and the item CF method for personalized recommendation based on user behavior. User CF is a method for searching similar users based on user history information. Its similarity calculation mainly selects Pearson correlation coefficient and cosine similarity as the main method. The formula of Pearson correlation coefficient is

(1) $$\begin{array}{c} s\left(u,v\right)=\frac{\sum_{i\notin I_{u}\cap I_{v}}\left(r_{u,i}-{\bar{r}}_{u}\right)\left(r_{v,i}-{\bar{r}}_{v}\right)}{{\sqrt{\sum_{i\notin I_{u}\cap I_{v}}\left(r_{u,i}-{\bar{r}}_{u}\right)}}^{2}\sqrt{\sum_{i\notin I_{u}\cap I_{v}}{\left(r_{v,i}-{\bar{r}}_{v}\right)}^{2}}}. \end{array}$$

The item is represented by *i*, *u* and *v* represent the user, *I*_*u*_ and *I*_*v*_, respectively, represent the evaluation item set of two users, *r*_*u*_ and *r*_*v*_, respectively, represent the average score of two users, *r*_*u*,*i*_ represents the score of user *u* on item *i*, and *r*_*v*,*i*_ represents the score of user *v* on item *i*. The cosine similarity formula is

(2) $$\begin{array}{c} s\left(u,v\right)=\frac{r_{u}\cdot r_{v}}{{\left\|r_{u}\right\|}_{2}{\left\|r_{v}\right\|}_{2}}. \end{array}$$

In addition, it is also necessary to calculate the prediction score of user *u* on the products without scoring. The prediction score of user *u* on the item *i* is

(3) $$\begin{array}{c} p_{u,i}={\bar{r}}_{u}+\frac{\sum_{u^{\prime }\in N}s\left(u,u^{\prime }\right)\left(r_{u^{\prime },i}-{\bar{r}}_{u}^{\prime }\right)}{\sum_{u^{\prime }\in N}\left|s\left(u,u^{\prime }\right)\right|}, \end{array}$$

where *s*(*u*, *u*′) represents the similarity between *u* and *u*′ users. At the same time, item CF is calculated in a similar way:

(4) $$\begin{array}{c} sim\left(i,j\right)={\text{corr}}_{i,j}. \end{array}$$

Then we can get

(5) $$\begin{array}{c} \text{sim}\left(i,j\right)={\text{corr}}_{i,j}=\frac{\sum_{u\in U}\left(R_{u,i}-{\bar{R}}_{u}\right)\left(R_{u,i}-{\bar{R}}_{u}\right)}{\sqrt{\sum_{u\in U}{\left(R_{u,i}-{\bar{R}}_{i}\right)}^{2}}\sqrt{\sum_{u\in U}{\left(R_{u,j}-{\bar{R}}_{j}\right)}^{2}}}, \end{array}$$

where *sim*(*i*, *j*) represents the similarity between *i* and *j*, and *corr*_*i*,*j*_ represents the correlation. Therefore, the user *u*'s predicted score *P*_*u*,*i*_ for item *i* is:

(6) $$\begin{array}{c} P_{u,i}=\frac{\sum_{j\in N}\text{sim}\left(i,j\right)\cdot r_{u,j}}{\sum_{j\in N}\left|\text{sim}\left(i,j\right)\right|}, \end{array}$$

where *N* represents the item set similar to item *i*, and *r*_*u*,*j*_ represents the user *u*'s score on item *j*.

### 3.2. Model of Improved K-Means Clustering Algorithm in Precision Marketing Mode of New Media

**Figure 3:**
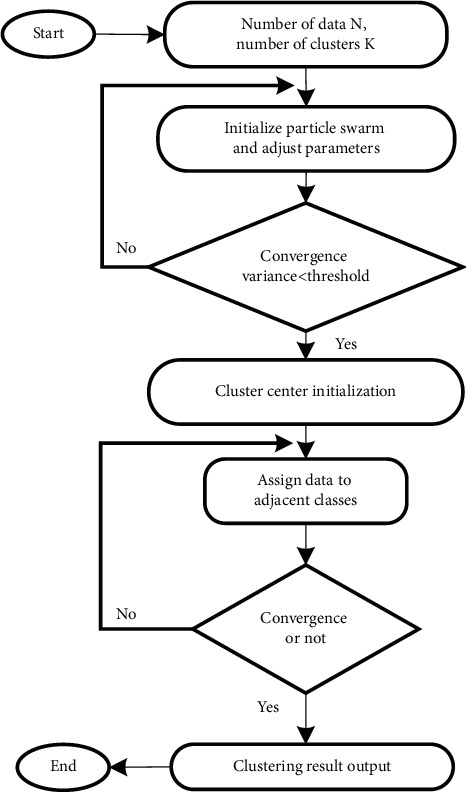
K-means algorithm flow.

K-means algorithm is widely used, and its idea is simple and easy to understand. And K-means algorithm is a clustering algorithm which is easy to implement. K-means algorithm has many advantages, such as simple idea and fast convergence speed. In the clustering analysis of large-scale data sets, the clustering algorithm is more efficient and excellent. In the data mining module of new media precision marketing system, this study uses K-means clustering algorithm to extract and classify the characteristics of customers. The algorithm flow is shown in Figure 3:

Clustering itself is to divide a data set into several data clusters, reduce the difference between data classes in the same data cluster as much as possible, and increase the difference between data classes in different clusters as much as possible (Xu *n* and others. 2021) [13]. Assuming that there is a set *A*={*a*_1_, *a*_2_…*a*_*n*_}, which is divided into *k* clusters {*c*_1_, *c*_2_ … *c*_*k*_}, there are

(7) $$\begin{array}{c} X=C_{1}\cup C_{2}\text{…}\cup C_{k}. \end{array}$$

*X* represents the result set after clustering, then it can further include

(8) $$\begin{array}{c} C_{i}\cup C_{j}=\emptyset \left(1\leq i,\;j\leq K,\;i\neq j\right). \end{array}$$

K-means algorithm calculates the distance between different data objects in the clustering process to judge the approximation between them. The clustering center can be described as

(9) $$\begin{array}{c} C=\left\{c_{j}|c_{j}=\left(c_{j1},c_{j2},\text{…}c_{jd}\right),\;j=1,2,\text{…}K\right\}. \end{array}$$

The Euclidean distance between *x*_*i*_ and *c*_*i*_ can be expressed as

(10) $$\begin{array}{c} Dis\left(x_{i},c_{j}\right)=\sqrt{\sum_{n=1}^{d}{\left(x_{in}-c_{jn}\right)}^{2}},\;i=1,2,\text{…},n;j=1,2,\text{…},K. \end{array}$$

*x*_*i*_ and *c*_*i*_ are defined as follows:

(11) $$\begin{array}{c} \left\{\begin{array}{c} x_{i}=\left(x_{i1},x_{i2},\text{…},x_{id}\right),\\ c_{j}=\left(c_{j1},c_{j2},\text{…},c_{jd}\right). \end{array}\right. \end{array}$$

In order to prevent the wrong selection of the initial clustering center in the clustering process, the particle swarm optimization algorithm is used to optimize the k-means algorithm. The particle swarm optimization algorithm is a method based on the group information transmission mode of bird swarm, which uses the information sharing of individuals in the group to convert the motion mode of the whole group from disordered motion to ordered motion in a certain solution space, so as to obtain the optimal solution of the problem (maihemuti *s* and others. 2021) [14]. A group contains a whole group of different individuals. These individuals are the particles in the particle swarm. The particles begin to fly at random speed in space. With the change of time, the position of the particles will change constantly. These particles that change their position continuously are the feasible solution of the problem. Find the best initial clustering center through the global search ability of particle swarm optimization algorithm. In the clustering process, it is necessary to calculate the average value of all data objects in each data class:

(12) $$\begin{array}{c} {\bar{x}}_{i}=\sum_{x\in C_{i}}\frac{x}{|C_{i}|}. \end{array}$$

Then the function value of the sum of squares of errors can be calculated:

(13) $$\begin{array}{c} E=\sum_{i=1}^{k}\sum_{x\in C_{i}}{\left|x-{\bar{x}}_{i}\right|}^{2}. \end{array}$$

The particle swarm search starts from the random position in space, and each particle starts to adjust itself according to the existing information, and then constantly approaches the global optimal solution. The search performance of this particle swarm can be judged by the fitness function of the current particle position. Based on formula (13), the fitness function of particle swarm optimization can be defined as

(14) $$\begin{array}{c} f\left(x\right)=\sum_{j=1}^{k}\left(\underset{x\in C_{j}}{\sum }\left\|X_{i}-C_{j}\right\|\right), \end{array}$$

where *f*(*x*) represents the fitness of particles, *X*_*i*_ represents the data sample, and *C*_*j*_ represents the cluster center. Because the two algorithms need to be converted in the clustering process, that is, the particle swarm optimization algorithm is used to find the initial clustering center of the data, and the k-means algorithm is used in the subsequent clustering analysis process, the two algorithms need to be switched, and the switching time is mainly measured by the convergence variance of the particle swarm optimization algorithm:

(15) $$\begin{array}{c} \sigma^{2}=\frac{1}{m{\sum_{i=1}^{m}\left[f\left(x_{i}\right)-f_{\text{avg}}\right]}^{2}}, \end{array}$$

where *σ* represents the convergence degree variance, *m* represents the particle swarm size, *f*(*x*_*i*_) represents the particle fitness, and *f*_avg_ represents the mean value of particle fitness. If the convergence degree variance reaches a very small state, it means that the particle swarm is still in the convergence state. Even if the particles are allowed to continue to iterate, a better solution cannot be obtained, and the redundant iteration range will waste computing time. Therefore, the best time for the system to change from particle swarm optimization algorithm to k-means algorithm is when particle swarm optimization reaches the convergence state. After data classification, users can be divided into different clusters according to different dimensions such as region, age and preference, and then personalized recommendation between customers, customer behavior, and customers can be carried out based on customer preference clusters.

## 4. Application Effect Analysis of New Media Precision Marketing Model for Traditional Media

### 4.1. Performance Analysis of Improved K-Means Clustering Algorithm

**Figure 4:**
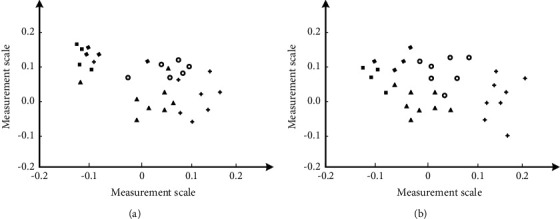
Effect drawing of initial cluster center. (a) k-means. (b) Improved k-means.

**Figure 5:**
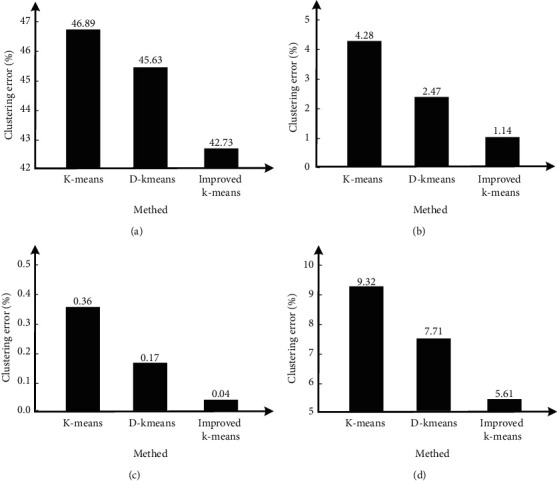
Comparative analysis of clustering errors. (a) Max. (b) Mean. (c) Min. (d) STD.

**Figure 6:**
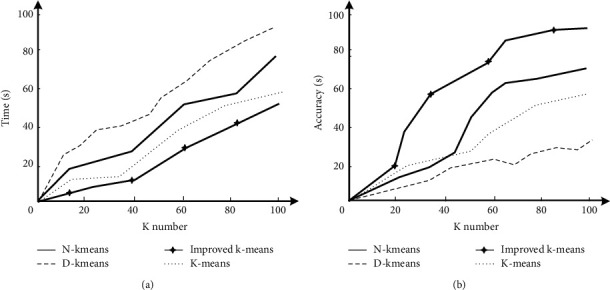
Comparative analysis of running time and accuracy of clustering error. (a) Run time comparison. (b) Comparison of transportation accuracy.

In the application experiment, this study analyzes the new media activities with the highest user preference. Two websites are randomly selected to discuss the impact of precision marketing system on users. This study analyzes the application effect of the new media precision marketing model for traditional media from two angles. The first angle is the performance analysis of the improved k-means clustering algorithm in this study, and the second angle is the application effect analysis of the new media precision marketing model for traditional media. In the analysis part of improved k-means clustering algorithm, firstly, the selection ability of improved k-means clustering algorithm for initial clustering center is analyzed, as shown in Figure 4:

Figure 4 shows the comparison of the initial clustering center selection effect between the traditional K-means algorithm and the improved k-means algorithm designed this time. It can be seen that although there are some obvious set categories in the overall distribution of the initial clustering center data points randomly selected by the traditional K-means algorithm, there are still individual data points in other data point sets, and this phenomenon occurs more frequently. Almost every data point category is mixed with other types of data points, and the category boundary between data points is not obvious. The initial clustering center data points selected by the improved k-means algorithm designed in this study form a set of data points with clear boundaries. There is almost no case that the data points are mixed between other types of data points, and the clustering effect is very obvious. It can be seen that the improved k-means algorithm in this study can reflect a stronger clustering effect when selecting the initial clustering center, and then obtain a high-quality initial clustering center. The selection performance of clustering center is much better than that of the unmodified k-means algorithm. The comparative analysis of clustering errors between different clustering algorithms is shown in Figure 5:

Figure 5 shows the comparison of clustering errors among the traditional K-means algorithm, d-means algorithm, and the improved k-means algorithm designed in this study. The clustering errors are compared from four aspects: the highest value, the lowest value, the average value, and the standard deviation. It can be seen that in the highest value, the clustering error of the traditional K-means algorithm is 46.89%, and the error of the improved k-means algorithm is 42.73%; At the lowest value, the clustering error of the traditional K-means algorithm is 4.28%, and the error of the improved k-means algorithm is 1.14%; On the average, the clustering error of the traditional K-means algorithm is 0.36%, and the error of the improved k-means algorithm is 0.04%; In terms of standard deviation, the clustering error of traditional K-means algorithm is 9.32%, which is the largest, while the error of improved k-means algorithm is 5.61%, which is the smallest. It can be seen that the clustering error of the improved k-means algorithm is the smallest of the three clustering algorithms in the four aspects of the highest value, the lowest value, the average value, and the standard deviation of the clustering error, and the clustering performance is the best. The comparison between running time and running accuracy is shown in Figure 6:

Figure 6 shows the comparison of running time and accuracy of n-kmeans, d-kmeans, K-means, and the improved k-means algorithm designed in this study. It can be seen that in terms of running time, with the increase of K value, the three running time broken lines show an overall upward trend, in which the position of K-means broken line is the highest, the interval is between 0s and 100s, and the fluctuation is the most frequent in the rising process, while the position of the improved k-means algorithm designed in this study is the lowest, the interval is between 0s and 60s, and the fluctuation is the most stable in the rising process, It can be seen that in terms of running time, the algorithm designed in this study takes less time and has a stronger performance; in terms of accuracy, the broken line position of the improved k-means algorithm in this study is the highest. After the K-value reaches 20, it enters an obvious rising period, and the accuracy finally reaches about 93%. It can be seen that the improved k-means algorithm has the strongest performance in running accuracy.

### 4.2. Application Effect Analysis of New Media Precision Marketing Model for Traditional Media

**Figure 7:**
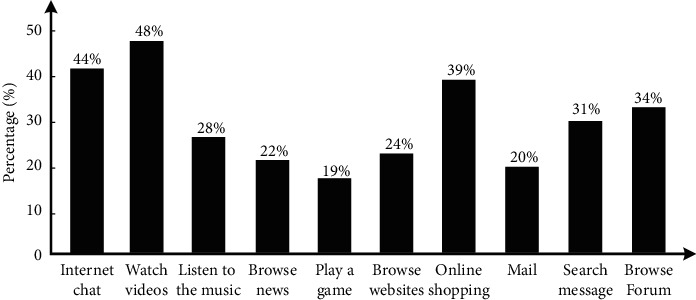
Network preference data.

**Figure 8:**
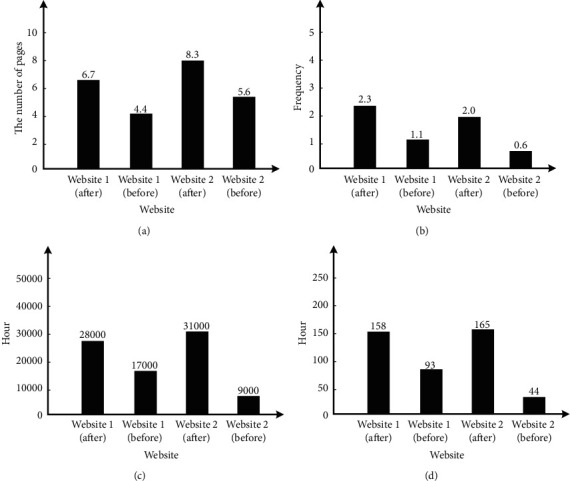
Website access data. (a) Page views per capita per day. (b) Per capita visits per day. (c) Average daily effective browsing time. (d) Per capita browsing time in a single day.

In the part of application effect analysis, this study is mainly divided into two parts: user preference data in new media activities and user access data on different websites. The preference data of users on the network is shown in Figure 7:

Figure 7 shows the preference performance of users in various new media activities. It can be found that the three activities with the strongest user preference in new media activities are watching video, online chat, and online shopping. Among them, 48% of users prefer watching video, 44% of users prefer online chat, and 44% of users prefer online shopping. When making personalized recommendation, it is suitable for advertising design and online recommendation from the above three aspects. Among them, the users who prefer to watch video are the most. Therefore, the recommendation in video is the most likely to attract the attention of users and has the highest marketing efficiency. Among all new media activities, online games, e-mail, and news browsing activities have lower preference. Among them, online game users account for 19%, e-mail users account for 20%, and news browsing users account for 22%, among which online game activities account for the lowest proportion, Therefore, when making marketing recommendations to users, advertising and marketing push in relevant activities should be avoided as far as possible, and the marketing efficiency of relevant activities is low. The analysis of access data for different websites is shown in Figure 8:

For all enterprises, whether traditional enterprises or Internet plus enterprises, the drainage and publicity of new media platforms is very important. If an enterprise can skillfully use new media marketing, it can attract traffic to the greatest extent and obtain the trust of intended customers, which is twice the result with half the effort for enterprise sales and publicity [15].

This study first numbers the website, and then analyzes the changes in its use of precision marketing system and the influence level of new media marketing on users.  Figure 8(a) shows the analysis of page views per capita in a single day. It can be seen that the page views per capita in a single day of website 1 increased from 4.4 to 6.7 after using the precision marketing system, while the page views per capita in a single day of website 2 increased from 5.6 to 8.3 after using the precision marketing system; Figure 8(b) shows the analysis of per capita visits per day. It can be seen that the per capita visits per day of website 1 increased from 1.1 to 2.3 after using the precision marketing system, while the per capita visits per day of website 2 increased from 0.6 to 2.0 after using the precision marketing system; Figure 8(c) shows the analysis of daily effective browsing time. It can be seen that the daily effective browsing time of website 1 increases from 17000 hours to 28000 hours after using the precision marketing system, while the per capita visits of website 2 in a single day increase from 9000 hours to 31000 hours after using the precision marketing system; Figure 8(d) shows the analysis of the per capita effective browsing time in a single day. It can be seen that the per capita effective browsing time in a single day of website 1 increases from 93 hours to 158 hours after using the precision marketing system, while the per capita effective browsing time in a single day of website 2 increases from 44 hours to 165 hours after using the precision marketing system. To sum up, the four indicators used in this analysis have increased after the adoption of the new media precision marketing system. Compared with website 1, website 2 has a greater growth rate, and the data of both websites have increased. It can be seen that the precision marketing system designed in this study has certain universality.

## 5. Conclusion

As a new form of media, new media can provide accurate marketing solutions for traditional media from the perspective of Internet of things data technology and promote the development of traditional media. Aiming at the shortcomings of K-means algorithm, this study improves k-means algorithm from the perspective of initial clustering center and particle swarm optimization algorithm, and combines it as a data mining tool and collaborative filtering algorithm as a personalized recommendation tool. On this basis, a new media precision marketing system is designed for traditional media, namely traditional media customer data mining and personalized recommendation marketing. The results show that the improved k-means algorithm is better than similar algorithms in clustering error and running time, its accuracy finally reaches about 93%, and the overall performance is also significantly better than other algorithms. In the application test, the new media precision marketing system successfully found the user's favorite new media activity, namely video viewing, accounting for 48%. After using the new media precision marketing system, the impact of the two websites on users has increased significantly. It can be seen that the system designed in this study has certain effectiveness and universality. However, this study also needs to simulate and verify the proposed optimization algorithm, and this part needs to be further elaborated in future research.

## Data Availability

The data used to support the findings of this study are available from the corresponding author upon request.

## References

[1] Peiulis Y. (2021). TV media change in the aspect of remediation theory. *Informacijos Mokslai*.

[2] Topbaş B. (2021). Adaptation of traditional media organs in video content: new media example: ct. *Turkish Online Journal of Design Art and Communication*.

[3] Lu J., Su D. K., Ro Y., Kim H. G. (2019). New media art research focused on tactile symbols. *TECHART Journal of Arts and Imaging Science*.

[4] Nazmine K. A., Chishti K. Z., Hannan K. T. (2021). New media technologies and society: a study ON the impact OF new media technology ON interaction patterns OF youth. *Tianjin Daxue Xuebao (Ziran Kexue yu Gongcheng Jishu Ban)/Journal of Tianjin University Science and Technology*.

[5] Ma T. (2019). Identification of new media technology factors and research on big data in the tourism industry. *ICIC Express Letters*.

[6] Wang R. (2021). Computer-aided interaction of visual communication technology and art in new media scenes. *Computer-Aided Design and Applications*.

[7] Orlando Morais F., Andriani K. F., Da Silva J. L. F. (2021). Investigation of the stability mechanisms of eight-atom binary metal clusters using DFT calculations and k-means clustering algorithm. *Journal of Chemical Information and Modeling*.

[8] Sudarsono B. G., Lestari S. P. (2021). Clustering pbyummm K-means. *JURNAL MEDIA INFORMATIKA BUDIDARMA*.

[9] Muhajir M., Ismail N., Syahreza S., Simanjuntak A. V. H. (2021). Pemutakhiran zmpam data blending b non-h K-means clustering. *Jurnal Fisika Flux: Jurnal Ilmiah Fisika FMIPA Universitas Lambung Mangkurat*.

[10] Sintiya S., Laksana T. G., Tanjung N. A. F. (2021). Kombinasi single linkage d K-means clustering upwdkp. *Journal of Innovation Information Technology and Application (JINITA)*.

[11] Qureshi I. H., Zahoor S. Z. (2018). Social media marketing and brand equity: a literature review. *Social Science Electronic Publishing*.

[12] Han X., Wang Z., Xu H. J. (2021). Time-weighted collaborative filtering algorithm based on improved mini batch K-means clustering. *Materials, Computer Engineering and Education Technology*.

[13] Xu N., Finkelman R. B., Dai S., Xu C. M. (2021). Average linkage hierarchical clustering algorithm for determining the relationships between elements in coal. *ACS Omega*.

[14] Maihemuti S., Wang W., Wang H., Wu J. (2021). Voltage security operation region calculation based on improved particle swarm optimization and recursive least square hybrid algorithm. *Journal of Modern Power Systems and Clean Energy*.

[15] Fajriansyah G. (2021). Analisis daftar pemilih tetap pada hasil rekapitulasi KPU berdasarkan usia menggunakan algoritma K-means (studi kasus:kota bandar lampung). *Electrician*.

